# Altered purinergic receptor expression in the frontal cortex in schizophrenia

**DOI:** 10.1038/s41537-022-00312-1

**Published:** 2022-11-14

**Authors:** Rawan Alnafisah, Anna Lundh, Sophie M. Asah, Julie Hoeflinger, Alyssa Wolfinger, Abdul-rizaq Hamoud, Robert E. McCullumsmith, Sinead M. O’Donovan

**Affiliations:** 1 Department of Neurosciences, University of Toledo, Toledo, OH USA; 2 Neurosciences Institute, Promedica, Toledo, OH USA

**Keywords:** Molecular neuroscience, Schizophrenia

## Abstract

ATP functions as a neurotransmitter, acting on the ubiquitously expressed family of purinergic P2 receptors. In schizophrenia (SCZ), the pathways that modulate extracellular ATP and its catabolism to adenosine are dysregulated. However, the effects of altered ATP availability on P2 receptor expression in the brain in SCZ have not been assessed. We assayed P2 receptor mRNA and protein expression in the DLPFC and ACC in subjects diagnosed with SCZ and matched, non-psychiatrically ill controls (*n* = 20–22/group). P2RX7, P2RX4 and male P2RX5 mRNA expression were significantly increased (*p* < 0.05) in the DLPFC in SCZ. Expression of P2RX7 protein isoform was also significantly increased (*p* < 0.05) in the DLPFC in SCZ. Significant increases in P2RX4 and male P2RX5 mRNA expression may be associated with antipsychotic medication effects. We found that P2RX4 and P2RX7 mRNA are significantly correlated with the inflammatory marker SERPINA3, and may suggest an association between upregulated P2XR and neuroinflammation in SCZ. These findings lend support for brain-region dependent dysregulation of the purinergic system in SCZ.

## Introduction

Adenosine triphosphate (ATP) functions as a neurotransmitter^1,2^, co-released with other neurotransmitters including serotonin and glutamate, to act on the extensive family of purinergic P2 receptors^3,4^. Upon release, extracellular ATP is rapidly catabolized to adenosine via a cascade of ectonucleotidase enzymes^5^. In schizophrenia (SCZ), the expression and activity of ectonucleotidases is significantly reduced in a region- and cell-subtype specific manner^6–8^. Downregulation of this enzymatic pathway that modulates the amount of time extracellular ATP spends in the synapse prior to its degradation may have a significant impact on P2R activation^6,8^. However, little is known about the expression of these receptors in the brain in SCZ.

There are seven P2X ionotropic receptors (P2X_1–7_) that bind ATP and eight P2Y metabotropic receptor subtypes (P2Y_1, 2, 4, 6, 11–14_) that bind ATP and other nucleotides (ADP and uridine di- and triphosphate (UDP, UTP)). P2 receptors are expressed throughout the brain^9–14^, with different P2 receptor subtypes found on neurons and glial cells including microglia, astrocytes and oligodendrocytes^13,15,16^. P2RX1–6 receptors form functional homotrimeric or heterotrimeric receptors^17^. P2RX7 typically forms homotrimers or functional heterotrimers with P2RX4^18^. It has yet to be determined which form of P2RX7 is predominant in the human brain^19^. P2RY receptors including P2RY12 also form homo- and hetero-oligomers^20–22^.

Perturbation of the purinergic system is implicated in the pathophysiology of psychiatric disorders like SCZ^23^. Purinergic signaling via P2 receptors is implicated in neuromodulation, intercellular communication, and energy metabolism^24–28^. ATP also serves as a danger associated molecular pattern (DAMP), and activation of P2Rs is an important regulator of neuroinflammation^25^. Activation of P2X receptors like P2RX7, which occurs only in the presence of elevated (micromolar range) ATP levels, induces pro-inflammatory cytokine release via activation of the NOD-, LRR- and pyrin domain-containing protein-3 (NLRP3) inflammasome^29–31^. Metabotropic P2RY receptor activation also results in changes in intracellular cAMP or Ca^2+^ concentrations, activating intracellular signaling cascades that regulate neuroinflammatory processes^25,32^. Despite a resurgence in interest in purinergic system dysregulation in SCZ^15,24,33^, our understanding of the expression and localization of P2 receptors in the brain in this disorder is limited. To address this gap in our knowledge, in this study, we assess mRNA and protein expression of P2RX and P2RY receptors in two different frontocortical brain regions in subjects diagnosed with SCZ.

## Methods

### Subjects

Dorsolateral prefrontal cortex (DLPFC, Brodmann area 9) samples from non-psychiatrically ill (*n* = 20–22) and SCZ subjects (*n* = 20–22) were obtained from the Maryland Brain Collection (MBC). Anterior cingulate cortex (ACC, Brodmann area 32) samples from non-psychiatrically ill (*n* = 20) and SCZ subjects (*n* = 20) were obtained from the Mount Sinai NIH Brain and Tissue Repository (NBTR). Subject demographics are described in Table 1 and Table S1. All cases were obtained with consent from the next of kin with IRB approved protocols and were diagnosed by two independent psychiatrists using DSM-IV diagnosing standards, based on review of available medical records, autopsy reports, and interviews with the family. Medication status was deemed “on” if the subjects were on antipsychotic medication in the 6 weeks prior to the end of life (NBTR) or based on postmortem toxicology analysis (MBC). Subjects were matched for age, sex, race, pH and postmortem interval (PMI) including for secondary analyses conducted in male and female groups (Table S2).

**Table 1:** Subject demographics.

	DLPFC				ACC	
	*qPCR study*		*Western immunoblot study*		*qPCR study*	
	CTL	SCZ	CTL	SCZ	CTL	SCZ
N	20	20	22	22	20	20
Age	41.95 ± 9.26	44.65 ± 9.38	42.73 ± 9.03	43.77 ± 10.44	78.25 ± 6.78	75.40 ± 7.84
Sex	10 M/10 F	10 M/10 F	13 M/9 F	13 M/9 F	12 M/8 F	11 M/9 F
Race	14 W/6B	13 W/7B	14 W/8B	12 W/10B	17 W/2H	19 W/1B
pH	6.54 ± 0.35	6.60 ± 0.43	6.66 ± 0.28	6.64 ± 0.40	6.57 ± 0.52	6.27 ± 0.23
PMI (hrs)	12.45 ± 4.99	13.65 ± 6.10	13.45 ± 5.19	15.41 ± 6.24	12.15 ± 6.92	13.10 ± 5.80
Antipsychotic Medication	N/A	6on/3off/11unk	N/A	6on/4off/12unk	N/A	12on/6off/2unk

Data presented as mean ± standard deviation.

*DLPFC* dorsolateral prefrontal cortex, *ACC* anterior cingulate cortex, *CTL* control, *SCZ* schizophrenia, *PMI (hrs)* postmortem interval in hours, *M* male, *F* female, *W* white, *B* black, *H* Hispanic, *N/A* not applicable, *N* subject number, *unk* unknown medication status.

### Quantitative polymerase chain reaction (qPCR)

Samples were prepared for qPCR analysis as previously described^8,34–36^. Briefly, DLPFC and ACC tissue blocks were cryo-sectioned (14 µm) onto glass slides (Superfrost Plus glass slides, Fisher Scientific). RNA was extracted from tissue sections with the RNeasy Mini Kit (#74134, Qiagen) as directed by the manufacturer’s instructions. Complementary DNA (cDNA) was synthesized using the High-Capacity cDNA Reverse Transcription Kit (#4368814, ThermoFisher Scientific) then diluted 1:3. QPCR was performed using SYBR-Green and Taqman primers (Table S3), in 96-well optical reaction plates (MicroAmp Fast Optical 96-well Reaction Plate, ThermoFisher Scientific) on a StepOne Real-Time PCR System (Applied Biosystems) for 3 min at 95 °C, 15 s at 95 °C for 40 cycles, and 1 min at 59 °C. Each 20 μL reaction included 3 μL of cDNA, 10 μL of SYBR-Green PowerUp Master Mix (ThermoFisher Scientific) and 3 pmol of each primer (Invitrogen, ThermoFisher Scientific). All samples were run in duplicate. Non-template (no cDNA) and no RT controls (template generated without reverse transcriptase enzyme) controls were run on all plates. Primers were designed based on previously published sequences (Table S3) or using Primerblast. All primers were tested by running PCR product on a 2% agarose gel and sequencing to confirm primer specificity^35^. Samples were normalized to a standard curve consisting of a pool of all samples. Data were normalized to the geometric mean of four reference genes: B2M, GAPDH, ACTB, and PPIA, whose expression was not significantly altered between groups.

### Immunoblotting

Western immunoblot was used to assay P2RX7 and P2RX4 protein expression, as previously described^37,38^. Briefly, twenty-five micrograms protein were run on 4–12% Bis-Tris gels (NuPAGE Invitrogen, ThermoFisher Scientific) for 1 h at 180 V. Following semi-dry transfer (18 V, 30 min) and 1 hr blocking (Licor blocking buffer) at room temperature, PVDF membranes were incubated at 4 °C overnight in primary antibody: rabbit anti-P2RX7 (1:1000, APR-004, Alamone), rabbit anti-P2RX4 (1:1000, APR-002, Alamone), goat anti-P2RX7 (1:1000, NBP1–37775, Novus) or a reference protein rabbit anti-valosin containing protein (VCP, 1:1000, ab109240, Abcam), diluted in blocking buffer (Licor) + 0.2% Tween20. Membranes were washed three times in TBS-T for 10 min, then incubated with anti-rabbit (1:1000, #68073, Licor) or anti-goat (1:1000, #32214, Licor) IR-dye labeled secondary antibodies diluted in blocking buffer (Licor) + 0.2% Tween + 0.01% SDS for 1 h at room temperature in the dark. Membranes were scanned using the LI-COR Odyssey laser-based imaging system. Band intensity values with segment median intra-lane background subtraction were determined using Image Studio v4.0. Near-infrared fluorescence value for each target protein was normalized to the in-lane value of VCP, and the normalized ratio from duplicate lanes was averaged. There was no changes in raw intensity values for VCP between the SCZ and CTL groups as we have previously reported^39^.

### Antipsychotic medication study

All experimental protocols were approved by the University of Alabama-Birmingham. Adult male Sprague-Dawley rats (250 g) were housed in pairs and maintained a 12 h light/dark cycle. To assess the effects of chronic antipsychotic administration, rats were randomly assigned to receive 28.5 mg/kg haloperidol-decanoate or vehicle (sesame oil) via intramuscular injection, once every 3 weeks for 9 months. Haloperidol-decanoate was used as a representative typical antipsychotic as most SCZ subjects for whom medication data was available were on typical antipsychotics at time of death. The brains were flash frozen on dry ice and stored at −80 °C until further use.

### Rat qPCR

Rat frontal cortex samples were prepared and assayed for qPCR as described above. Rodent primers are listed in Table S3.

### In Silico analysis

A “look-up study” of purinergic receptor gene expression in postmortem brain tissue in SCZ subjects who were “on” and “off” antipsychotic medications was conducted using the Stanley Medical Research Institute (SMRI) Online Genomics Database^40^. The fold change and *p* value for selected genes are listed in Table 2. A radar chart showing the relative proportion of P2R gene expression in different human brain cell types (BrainAtlas, accessed from Kaleidoscope^41^) was generated using Excel v2207.

**Table 2:** The Stanley Medical Research Institute (SMRI) Online Genomics Database reports P2R gene expression in SCZ subjects who were “on” compared to “off” antipsychotic medication at time of death.

SCZ on/off antipsychotic medication from SMRI		
Gene Symbol	Fold change	*P* value
*P2RX4*	1.13	0.003
*P2RX5*	1.01	0.701
*P2RX7*	1.01	0.555

The fold change in mRNA expression and p-values of selected genes *P2RX4, P2RX5, P2RX7* from SMRI dataset are presented.

### Data analysis

All data were tested for normal distribution using the D’Agostino and Pearson test, and for variance using F-test. Outliers two or more standard deviations from the mean were excluded. Data was log transformed if not normally distributed. Rat data was normal and was not log transformed. Regression analysis was performed to determine associations between transcript or protein expression and age, PMI or pH value. If no significant associations were found, data were analyzed using Student’s *t* test (parametric), Welch’s *t* test (unequal variance) or Mann-Whitney U test (non-parametric). If significant associations were found, data were analyzed using analysis of covariance (ANCOVA). The association between P2RX and SERPINA3 mRNA expression was assessed using Spearman’s rho. Data were analyzed using Graphpad Prism v8.0.2 (Graphpad) and Statistica v13.3 (Statsoft). Alpha <0.05 for all tests.

## Results

### Purinergic receptor gene expression in schizophrenia

The relative gene expression levels of purinergic receptors *P2RX4*, *P2RX5*, *P2RX7*, *P2RY12*, and *P2RY13* were assayed in the DLPFC and ACC in SCZ. These targets were selected based on their association with psychiatric disorders^42–47^ as well as their expression pattern in the human brain, which was determined using Genotype-Tissue Expression (GTEx) and Brain-RNAseq (https://www.brainrnaseq.org/) (Fig. S1)^48,49^.

**Fig. 1:**
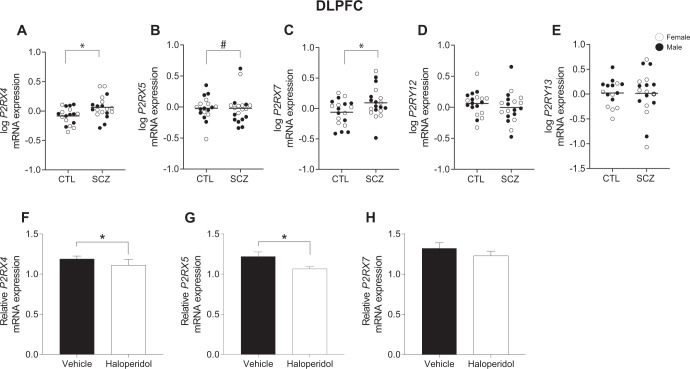
Purinergic receptor gene expression in the DLPFC in SCZ. *P2RX4* (**A**) and *P2RX7* (**C**) gene expression were significantly increased (*p* < 0.05) in SCZ subjects compared to CTL. (**B**) P2RX5 mRNA expression was significantly reduced (^#^*p* < 0.05) in male SCZ subjects compared to CTLs. There was no significant difference in *P2Y12* or *P2Y13* mRNA expression (**D**, **E**). Data presented as mean, *n* = 16–20/group, (**F–H)** Purinergic receptor gene expression in the frontal cortex of rats administered chronic haloperidol-decanoate. There was a significant decrease in *P2RX4* (**F**) and *P2RX5* (**G**) mRNA expression in antipsychotic-treated rats administered haloperidol-decanoate compared to vehicle-treated controls. There was no significant difference in *P2RX7* (**H**) gene expression. Data presented as mean ± SEM, *n* = 9–10/group. *p < 0.05. CTL control, DLPFC dorsolateral prefrontal cortex, SCZ schizophrenia.

In the DLPFC, mRNA expression of *P2RX4* (*t* = 2.9, *p* = 0.006, *n* = 18–20/group) and *P2RX7* (*t* = 2.091, *p* = 0.0436, *n* = 19/group) was significantly increased in SCZ (Fig. 1A, C). *P2RX5* mRNA levels were significantly reduced (*t* = 2.37, *p* = 0.0299, *n* = 9–10/group) in male SCZ subjects compared to same-sex CTLs (Fig. 1B). There was no significant difference in mRNA levels of *P2RY12* or *P2RY13* in the DLPFC (Fig. 1D, E) or in purinergic receptor mRNA levels in the ACC (Fig. S2). There was no significant association between pH, PMI or age and purinergic receptor gene expression. Correlation plots for age, which is older in the ACC cohort (mean 76.8 ± 7.37) than the DLPFC cohort (mean 43.4 ± 9.3), and P2R expression are shown in Fig. S3.

### Effects of antipsychotics on purinergic receptor expression

To account for the potential effects of chronic antipsychotic treatment on expression of significantly altered purinergic receptor transcripts in the brain, we assayed *P2rx4*, *P2rx5* and *P2rx7* mRNA levels in the frontal cortex of rats treated for 9 months with haloperidol-decanoate. There was a significant decrease in mRNA levels of *P2rx4* (*p* = 0.0118, *t* = 2.820, *n* = 9–10/group) (Fig. 1F). However, “look-up” studies using the SMRI Online Genomics Database found a significant increase (*p* = 0.003, fold change = 1.13) in *P2RX4* expression in SCZ “on” antipsychotic subjects compared to SCZ “off” antipsychotic subjects (Table 2), suggesting a disease-drug interaction that is not found in rodent models of antipsychotic administration. There was a significant decrease in *P2rx5* (*p* = 0.024, *t* = 2.46, *n* = 10/group) in rats administered haloperidol-decanoate compared to vehicle (Fig. 1G). “Look-up” studies show no significant difference (*p* > 0.05) in *P2RX5* in SCZ subjects “on” compared to “off” antipsychotics (Table 2). There was no significant difference in mRNA levels of *P2RX7* in haloperidol-decanoate treated rats or in “look-up” studies (Fig. 1H, Table 2).

### P2RX7 protein expression in the DLPFC in SCZ

**Fig. 2:**
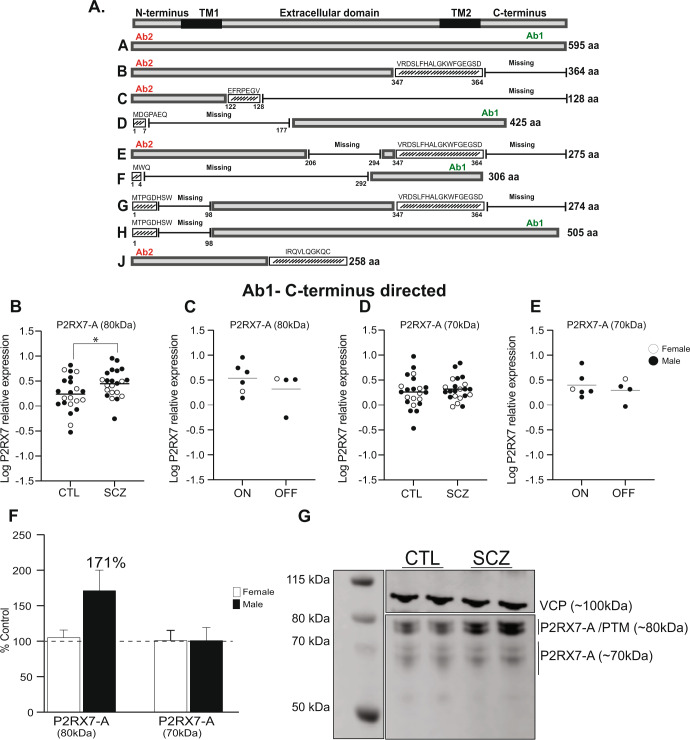
P2RX7 protein expression in the DLPFC. **A** Schematic representation of the human P2RX7 receptor protein isoforms. P2RX7-A is the full-length (595 amino acid), canonical isoform. Isoforms P2RX7-B –J are truncated or contain alternative sequences. To detect P2RX7-A expression a C-terminus directed antibody (Antibody 1(Ab1)) was used. The expression of other P2RX7 isoforms were assayed using an N-terminus domain directed antibody (Ab2). **B** Protein expression of the P2RX7-A 80 kDa band is significantly increased (p < 0.05) in SCZ compared to CTL, using Ab1. **C** There was no significant difference in P2RX7-A 80 kDa protein expression in SCZ subjects who were “on” vs. “off” antipsychotic medication. **D** There was no significant difference in P2RX7-A 70 kDa isoform expression or (**E**) in SCZ subjects who were “on” vs. “off” antipsychotic medication. **F** Relative expression (% control) of P2RX7-A 80 kDa and 70 kDa in female (open bars) and male (black bars) SCZ subjects. Data presented as mean ± SEM. **G** Representative image of P2X7R immunoblot using C-terminus directed antibody (Ab1). P2RX7-A forms doublet bands at approximately 80 kDa (potential N-glycosylated form) and 70 kDa (non-post translationally modified). SCZ and CTL samples were run in duplicate for each subject. VCP protein control is expressed at approximately 100 kDa. Data presented as mean, blots were analyzed by Student’s *t* test *p < 0.05, *n* = 22/group, CTL vs. SCZ. DLPFC dorsolateral prefrontal cortex, SCZ schizophrenia, CTL non-psychiatrically ill controls, VCP valosin containing protein.

Using a knockout-validated P2RX7 C-terminus directed antibody (Ab1) (Fig. 2A), we identified an 80kDA band, likely corresponding to the N-glycosylated form of P2RX7-A isoform^50,51^, that was significantly increased (*t* = 2.1, *p* = 0.03, *n* = 22/group) in SCZ subjects compared to CTLs (Fig. 2B). Glycosylated P2RX7-A was previously reported at ~80 kDa in human^50–52^ and mouse^53^ models. There was no significant difference in 80 kDa P2RX7-A expression in SCZ subjects who were “on” and “off” antipsychotic medication (Fig. 2C). Conversely, no significant difference in the canonical 70 kDa P2RX7-A expression was found between SCZ and CTLs (*t* = 0.6, *p* = 0.49, *n* = 22/group, Fig. 2D) or in SCZ subjects who were “on” and “off” antipsychotic medication (Fig. 2E).

There was no significant difference in P2RX7-A 80 kDa in female (*t* = 1.004, *p* = 0.33, *n* = 9/group) or male (*t* = 1.92, *p* = 0.065, *n* = 9/group) SCZ subjects relative to same-sex CTLs, although significant increases in P2RX7-A (80 kDa) appear to be driven primarily by male subjects (171% increase relative to CTL, Fig. 2F). There was no significant sex difference in P2RX7-A (70 kDa) expression. Representative immunoblots of Ab1 P2RX7 isoform expression shown in Figs. 2G, S4.

To determine if expression of the C-terminus truncated isoforms of P2RX7 (isoforms -B, -C, -E, -J) that are reportedly expressed in human tissues^54–56^ are altered in the brain in SCZ, we used an alternative N-terminus directed antibody, Ab2 (Fig. 2A). P2RX7-C, -E and -J are non-functional i.e., incapable of forming a channel receptor and therefore fail to activate different biological processes^52,55^. P2RX7-B forms a functional ion channel but not a macropore, so its activation does not induce cell lysis^50,57^. Expression of these non-canonical P2XR7 isoforms can affect the function of the receptor. P2RX7-J assembly with P2RX7-A forms a non-functional heteromeric receptor that may protect certain cell types from ATP-induced cell death, as reported in ocular and malignant epithelial cells^52,58^. It is still unclear how P2RX7 isoforms function in human brain, and whether their expression is altered in neuropsychiatric disorders including SCZ.

**Fig. 3:**
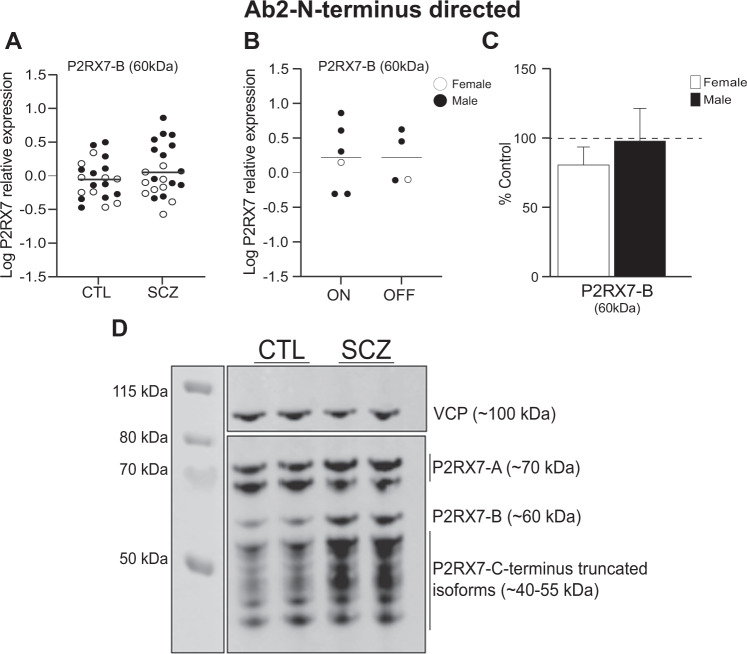
P2RX7 protein expression detected by N-terminus directed antibody (Ab2). **A** There was no significant difference in P2RX7-B 60 kDa bands protein expression in SCZ subjects compared to CTL. **B** There was no significant difference in P2RX7-B protein expression at 60 kDa band using Ab2 in SCZ subjects “on” vs. “off” antipsychotic medications. **C** Relative expression (% control) of P2RX7-A 60 kDa in female (white bars) and male (black bars) SCZ subjects. Data presented as mean ± SEM. **D** Representative images of P2RX7 immunoblot using N-terminus directed antibody. SCZ and CTL samples were run in duplicate for each subject. VCP protein control is expressed approximately 100 kDa. Data presented as mean, blots were analyzed by Student’s *t* test, *n* = 19–22/group. DLPFC dorsolateral prefrontal cortex, SCZ schizophrenia, CTL non-psychiatrically ill controls, VCP valosin containing protein.

We found no significant difference between CTL and SCZ (*t* = 0.9, *p* = 0.32, *n* = 21–22/group) subjects in the expression of the ~60 kDa band that likely corresponds to P2RX7-B^50^ (Fig. 3A) or in SCZ subjects who were “on” and “off” medication (Fig. 3B). There was no significant sex difference in P2RX7-A (70 kDa) expression (Student’s *t* test, *p* > 0.05) (Fig. 3C). As in our study, P2RX7-B expression has previously been reported to run at a higher than predicted (42 kDa) molecular weight^50^, although others report that P2RX7-B may be expressed as doublet bands (~42–45 kDa) in postmortem striatum tissue^10^. However, as little is known about the protein expression of P2RX7 in human brain, further studies will be required to confirm specific isoform expression in this tissue. Protein bands corresponding to P2RX7-C and P2RX7-E were not identified at the expected size (14 kDa and 31 kDa, respectively). These isoforms may not be expressed in the human brain^55,56^, or in the DLPFC, specifically. Alternatively, these isoforms may not be expressed at their predicted molecular weights. We identified a series of protein bands between 40–55 kDa (Fig. 3D). A similar pattern of expression was previously reported in postmortem human brain tissue using the same antibody^10^. These bands may represent different truncated P2RX7 isoforms or may be an artifact of the antibody (non-specific labeling). As the identity of these bands could not reliably be assigned to a P2RX7 isoform, they were not quantified in this study.

As with Ab1, doublet bands corresponding to the canonical P2RX7-A isoform were identified at ~70 kDa using Ab2 (Fig. 3D)^59^. However, no higher molecular weight (80 kDa) P2RX7-A bands were identified. Previous studies have reported that N-terminus directed Ab2 may be less sensitive for P2RX7-A relative to the C-terminus directed Ab1^10^. Thus, we utilized the data obtained from Ab1, which is a knockout-validated antibody, for quantification.

Overall, P2RX7 is expressed as multiple isoforms, forms homomeric and heteromeric receptor complexes and thus is expected to be detected in bands at different molecular weights by immunoblot. However, our understanding of the expression and localization of P2RX7 protein isoforms in human brain is still limited. Further studies will be required to confirm the expression of specific P2RX7 isoforms in different brain regions.

### P2RX4 protein expression in SCZ at the DLPFC

**Fig. 4:**
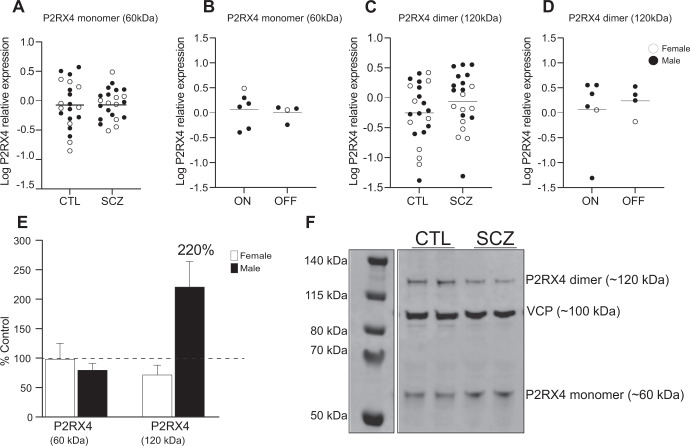
P2RX4 protein expression in the DLPFC in SCZ. **A** There was no significant difference in P2RX4 monomer (60 kDa) protein expression in SCZ compared to CTL subjects or (**B**) in SCZ subjects “on” vs. “off” antipsychotic medication. **C** There was no significant difference in P2RX4 dimer (120 kDa) in SCZ compared to CTL subjects or (**D**) in SCZ subjects who were “on” vs. “off” antipsychotic medications. **E** Relative expression (% control) of P2RX7-A 60 kDa in female (white bars) and male (black bars) SCZ subjects. Data presented as mean ± SEM. **F** Representative images of P2RX4 immunoblot. SCZ and CTL samples were run in duplicate for each subject. VCP protein control is expressed ~100 kDa. Data presented as mean, blots were analyzed by Student’s *t* test, *n* = 22/group. DLPFC dorsolateral prefrontal cortex, SCZ schizophrenia, CTL non-psychiatrically ill controls, VCP valosin containing protein.

There was no significant difference in P2RX4 isoform-1 monomer expression (~60 kDa) (*t* = 0.054, *p* = 0.95, *n* = 21–22/group, Fig. 4A) or in SCZ subjects who were “on” and “off” medication (Fig. 4B). There was also no significant difference in dimer expression (~120 kDa) (*t* = 1.16, *p* = 0.25, *n* = 21–22/group Fig. 4C) in SCZ compared to CTL subjects or in SCZ subjects who were “on” and “off” medication (Fig. 4D).There was no significant sex difference in P2RX4 monomer expression or female P2RX4 dimer expression. P2RX4 dimer expression was increased 220% in male SCZ subjects relative to same-sex CTLs (Fig. 4E) but this was not statistically significant (*p* = 0.0501). We observed no significant difference in lower molecular weight bands at 44 kDa (*t* = 0.18, *p* = 0.85, *n* = 21–20/group) corresponding to P2RX4 isoform-2, and at 40 kDa (*t* = 0.05, *p* = 0.95, *n* = 21–22/group) corresponding to P2RX4 isoform-3 (data not shown) (Fig. S4).

### P2RX4 and P2RX7 and neuroinflammation

**Fig. 5:**
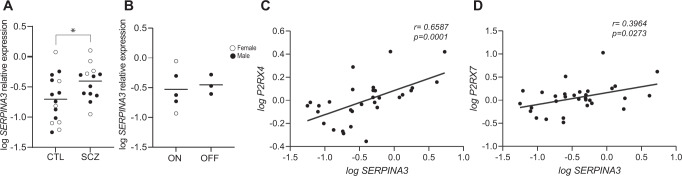
Association between P2RX and inflammatory markers in SCZ. **A** *SERPINA3* gene expression in the DLPFC was significantly increased (*p* < 0.05) in SCZ subjects compared to CTLs, *n* = 13–15/group. Data presented as mean, Student’s *t* test *p < 0.05. **B** Significant difference in *SERPINA3* expression in SCZ subjects who were “on” antipsychotic medications vs. CTL subjects (*t* = 2.08, *p* = 0.04, *n* = 10–15/group). Significant correlation (Spearman’s r, *p* < 0.05, *n* = 31) between *P2RX4* and *SERPINA3* mRNA (**C**) and (**D**) *P2RX7* and *SERPINA3* mRNA. Data presented as mean, *n* = 13–16/group. DLPFC dorsolateral prefrontal cortex, SCZ schizophrenia, CTL non-psychiatrically ill controls.

P2X receptor activation is associated with upregulated immune response. We assessed the association between mRNA expression of the inflammatory marker *SERPINA3* and *P2RX4* and *P2RX7* mRNA expression in the DLPFC. Increased *SERPINA3* mRNA expression is a robust marker of inflammation in SCZ^60,61^, and was significantly upregulated in this study in SCZ compared to CTL subjects (*t* = 2.26; *p* = 0.03, *n* = 13–15/group, Fig. 5A). There was no significant difference in *SERPINA3* expression in the “on” and “off” medication SCZ subjects (Fig. 5B). There was a significant positive association between mRNA expression of *P2RX4, P2RX7* and *SERPINA3* (Fig. 5C, D).

## Discussion

ATP is released into the extracellular milieu and sequentially hydrolyzed to adenosine via a series of extracellular enzymes^62^. In SCZ, we, and others, have reported significant changes in the pathways responsible for the extracellular catabolism of ATP in postmortem brain tissue^6,8,63,64^. However, little attention has been paid to how perturbations of ATP availability affects purinergic receptor expression^10,11,33^. Identifying the different isoforms and multimeric structures of P2Rs, many of which have not previously been identified in human brain, posed challenging. Thus, we focus our discussion on findings of the canonical P2XR isoforms detected using knockout-validated antibodies. Overall, our findings suggest disease-dependent changes in P2RX mRNA and protein isoform expression in the frontal cortex.

We identified significant increases (*P2RX4* and *P2RX7)* and decreases (*P2RX5)* in P2X receptor mRNA expression in the DLPFC in SCZ. Changes in *P2RX4* and *P2RX5* expression may be due to antipsychotic medication. “Look-up” studies of postmortem transcriptomic datasets of SCZ subjects who were “on” vs. “off” antipsychotics found increased *P2RX4* expression in SCZ subjects who were “on” medication. Conversely, in a rodent model, chronic haloperidol-decanoate administration resulted in significant reductions in *P2rx5* mRNA expression in the rat frontal cortex, suggesting that reduced *P2RX5* expression may be driven by antipsychotic medication effects in male SCZ subjects.

We also assessed the protein expression of P2RX4 and P2RX7, which were altered at the mRNA level in the DLPFC in SCZ. Using a knockout-validated antibody, we detected monomer (~60 kDa) and dimer (~120 kDa) bands of P2RX4 isoform-1 protein^65^. Although not statistically significant, increases in P2RX4 dimer expression were found in male but not female SCZ subjects relative to CTLs. Interestingly, injury-induced P2RX4 upregulation was previously found in male but not female mice in a spared nerve injury pain model^66^. P2RX4 is implicated in SCZ-associated behaviors. Sensorimotor gating is a form of CNS inhibition that filters unnecessary information so that attention is focused on salient information^67^. Deficits of sensorimotor gating, as measured by prepulse inhibition (PPI), is a robust endophenotype of SCZ^68^, although relatively few studies have been conducted in female patients^69^. Potentiation of the P2RX4 receptor, by the allosteric modulator ivermectin, disrupts PPI^70,71^, and deficits in PPI are also reported in *P2rx4* knockout mice^72^, supporting a role for this receptor in sensorimotor-gating deficits in SCZ. Additionally, recent studies suggest that P2RX4 stimulation leads to hyperactivity of dopamine transmission, which is implicated in the onset of SCZ symptoms^73^, and disruption of PPI^71^. P2RX4 receptor antagonism has been proposed as a potential therapeutic target to improve sensorimotor-gating deficits in disorders like SCZ^71^. Increases in P2RX4 expression may also reflect a response to elevated ATP and neuroinflammation found in the brain in SCZ^60^. P2RX4 activates the NLRP3 inflammasome and pro-inflammatory cytokine release associated with neuroinflammation^74^.

We report a similar change in P2RX7 expression in SCZ. The 80 kDa band, which likely corresponds to the glycosylated form of P2RX7-A^50–53^, is significantly increased, likely driven by changes in male SCZ subjects. N-glycosylation occurs at 5 different sites on P2RX7-A^75,76^, converting it into a fully mature and functional protein^51,77^. Glycosylation plays an important role in P2RX7-A receptor trafficking to the plasma membrane, localization, ATP sensing, channel formation, and pore activation^76,78–80^. Recent studies in the phencyclidine (PCP)-induced model of SCZ found that blocking P2RX7 alleviates SCZ-like behaviors including spatial memory impairment, hyperlocomotion, and social withdrawal^42,81^. Increasingly, P2RX7 is recognized as a regulator of neuroinflammation and a potential therapeutic target in neuropsychiatric disorders^82^. P2RX7 is relatively insensitive to ATP, requiring high levels (micromolar range) as occurs during injury or illness, to become sensitized and form a pore^83^. Consequently, P2RX7 stimulation can initiate multiple downstream events, including activation of pro-inflammatory cytokines interleukin-1β (IL-1β), interleukin-8 (IL-8), and interleukin-6 (IL-6). Reports of altered cytokine levels in SCZ are mixed, with no^84,85^ or elevated levels found^60,86^, although robust increases are consistently reported in a “high inflammatory” subset of SCZ subjects^60,87,88^.

**Fig. 6:**
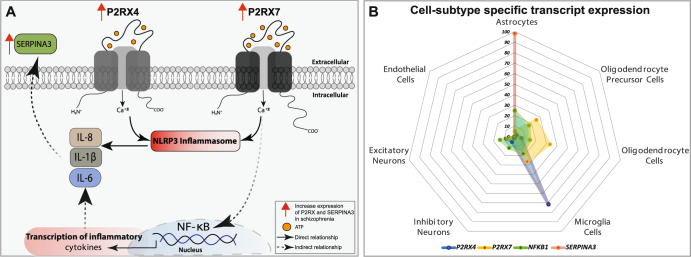
P2RX receptors and inflammatory markers in SCZ. **A** A proposed P2RX receptor pathway leading to inflammation in SCZ. In response to activation of P2RX4 or P2RX7, the NLRP3 inflammasome activated pro-inflammatory cytokines. P2XRs may also act via the NF-kB pathway, an established inflammatory pathway in SCZ. These inflammatory molecules contribute to neuroinflammation, as indicated by upregulated expression of inflammatory marker SERPINA3 in SCZ. Solid arrows show direct relationship between targets and dashed arrows represent indirect relationship between targets. **B** Radar chart demonstrating differential *P2RX4, P2RX7, NF-kB*, and *SERPINA3* gene expression in different cell types in human brain. CTL control, SCZ schizophrenia, DLPFC dorsolateral prefrontal cortex, IL interleukin, NLRP3 NOD-, LRR- and pyrin domain-containing protein-3, P2XR purinergic receptor, NF-kB nuclear factor kappa.

The role of P2RX receptors in the inflammatory response in SCZ has yet to be elucidated. We found a significant positive association between *P2RX4* and *P2RX7* and *SERPINA3* mRNA expression. SERPINA3 is a marker of neuroinflammation that is consistently increased in the brain in SCZ^60,87,89–91^, a finding that was replicated in this study. Models of NF-kB-driven cytokine release and SERPINA3 upregulation contributing to a neuroinflammatory state have been proposed in SCZ Fig. 6 ^60,84^. Interestingly, P2RX7 activation also stimulates the NF-kB pathway^92–94^, as well as the NLRP3 inflammasome, resulting in cytokine release and indirectly, SERPINA3 synthesis^61^. Our data suggest that increased purinergic receptor expression, particularly P2RX7, may also be associated with neuroinflammation in SCZ. Further studies are required to determine whether P2XR7-NLRP3 or P2RX7-NF-kB pathway activation serve as mechanisms for increased neuroinflammation found in SCZ.

Rodent models of antipsychotic medication administration provide a useful tool to understand drug effects on gene expression in the brain. However, they cannot fully recapitulate the effects of medication in a complex disease. Thus, we utilize a combination of animal model and postmortem “look-up” studies and statistical analysis, where feasible, to account for the effects of psychotropic medications on our dependent measures. While changes identified in P2R isoform expression do not appear to be an effect of antipsychotic medications, our analysis was limited to the subjects for whom postmortem toxicology data was available. Larger studies comparing protein expression in subjects who were “on” vs. “off” medication will be required to confirm this. The finding that *P2RX4* mRNA expression was differentially altered in SCZ subjects who were on/off medication and in antipsychotic-treated rat brain, however, suggests unique disease-drug interaction effects that can only be fully assessed in translational studies of disease. Alternatively, data obtained from the SMRI does not differentiate antipsychotic drug class and assignment of on/off antipsychotic medication is based on prescription and likely compliance^95^, which may also contribute to differences when compared with studies of haloperidol-treated rats.

Interestingly, our findings indicate potential sex differences in P2RX4 and P2RX7 isoform expression in SCZ. Secondary analyses of P2R expression in males and females were not statistically significant, however, effect size (presented here as % control) indicate that changes in P2RX protein isoform expression were driven by increases in male SCZ subjects. These findings are in line with reports of sex differences in SCZ; SCZ is more prevalent, develops at an earlier age, and symptoms are typically more severe in male compared to female subjects^38,96,97^. We also found a significant decrease in *P2RX5* mRNA expression in male SCZ compared to male CTLs. Although a similar decrease in *P2RX5* mRNA in our rodent model of chronic antipsychotic administration suggests that this change is likely an effect of medication, P2R mRNA expression was only assessed in male rats in this study. Previous studies have also reported sex-specific changes in P2Rs in SCZ, including a significant increase in *P2RY12* mRNA in male SCZ subjects who died by suicide^98,99^. As a result, the P2R system may play a role in sex-specific differences in the onset or severity of SCZ symptoms.

Overall, our study suggests brain-region and disease related changes in P2RX4 and P2RX7 receptor expression in SCZ. We applied animal model, in silico and statistical approaches to account for the potential effects of medication on these findings, although we cannot exclude that antipsychotics may play a role in some of these changes. Further work will be required to determine the expression and function of P2RX receptors and their various isoforms in different cell types in the human brain, and whether they are altered in disease. Increased P2R expression may contribute to different facets of SCZ pathophysiology including deficits in sensorimotor gating and increased neuroinflammation. These findings lend further support for perturbation of the purinergic system in the neurobiology of SCZ.

## Supplementary information

Supplementary Information
