# Supplementary material for: Altered purinergic receptor expression in the frontal cortex in schizophrenia
Source: Schizophrenia (Heidelb). 2022 Nov 14;8(1):96. doi: 10.1038/s41537-022-00312-1 (PMC9663420; doi:10.1038/s41537-022-00312-1)
Supplement: Supplementary file 1 — Supplementary Information [file 41537_2022_312_MOESM1_ESM.pdf]

Figure S1.

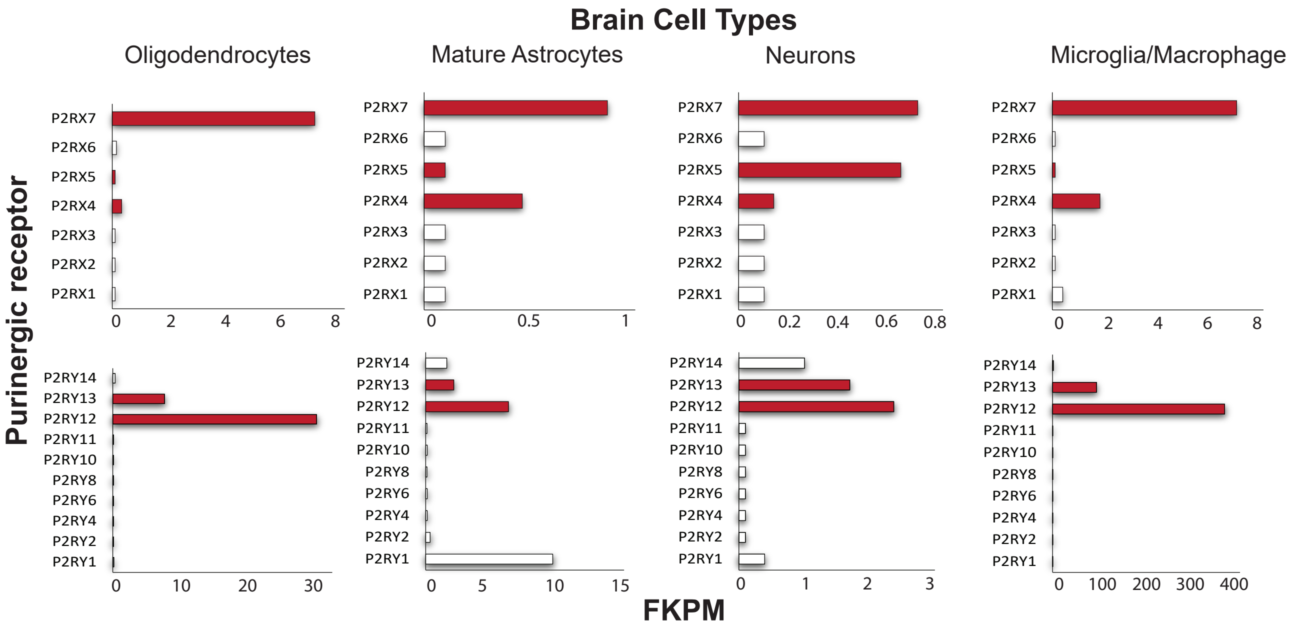

Figure S1. Relative gene expression (fragments per kilobase million (FPKM)) values of P2RX (upper panel) and P2RY (lower panel) receptors in human mature astrocytes, neurons, endothelial cells and oligodendrocytes. Data obtained from BrainRNAseq database. Purinergic receptors that are implicated in psychiatric disorders and that have relatively high levels of expression in the human brain were assayed in this study (red bars).

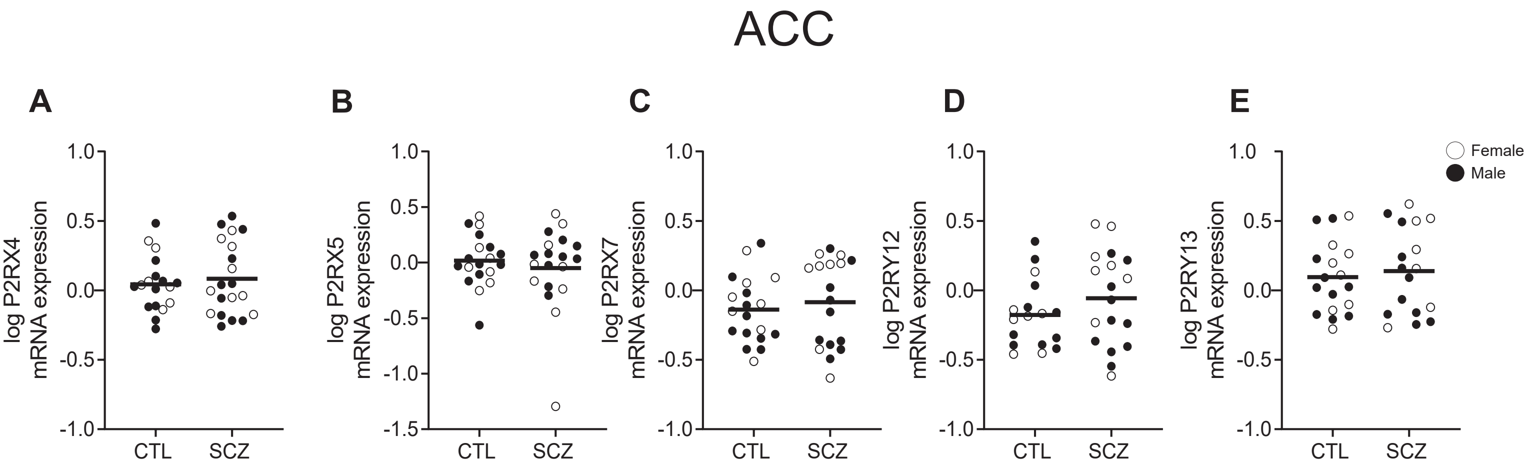

Figure S2. Purinergic receptor gene expression in the anterior cingulate cortex (ACC) in schizophrenia (SCZ) (A-E). There was no significant difference in gene expression of P2RX4-5, 7 or P2RY12-13 receptor gene expression in SCZ subjects compared to non-psychiatrically ill controls (CTL). Data presented as mean, n=17-20/group.

Figure S3.

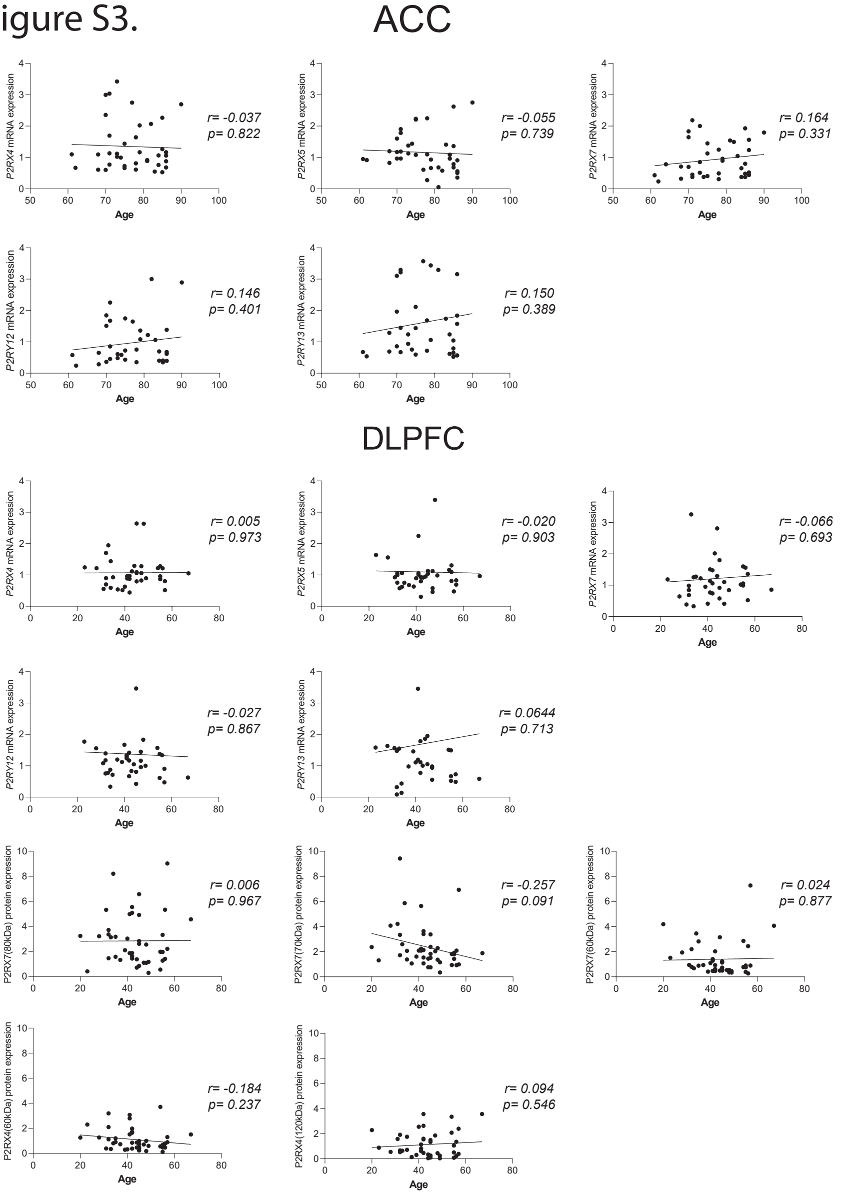

Figure S3: Association between the age and P2R mRNA and protein expression in ACC and DLPFC brain regions. There were no significant correlations between age and P2R expression. Pearson's r and p-value presented.

Figure S4.

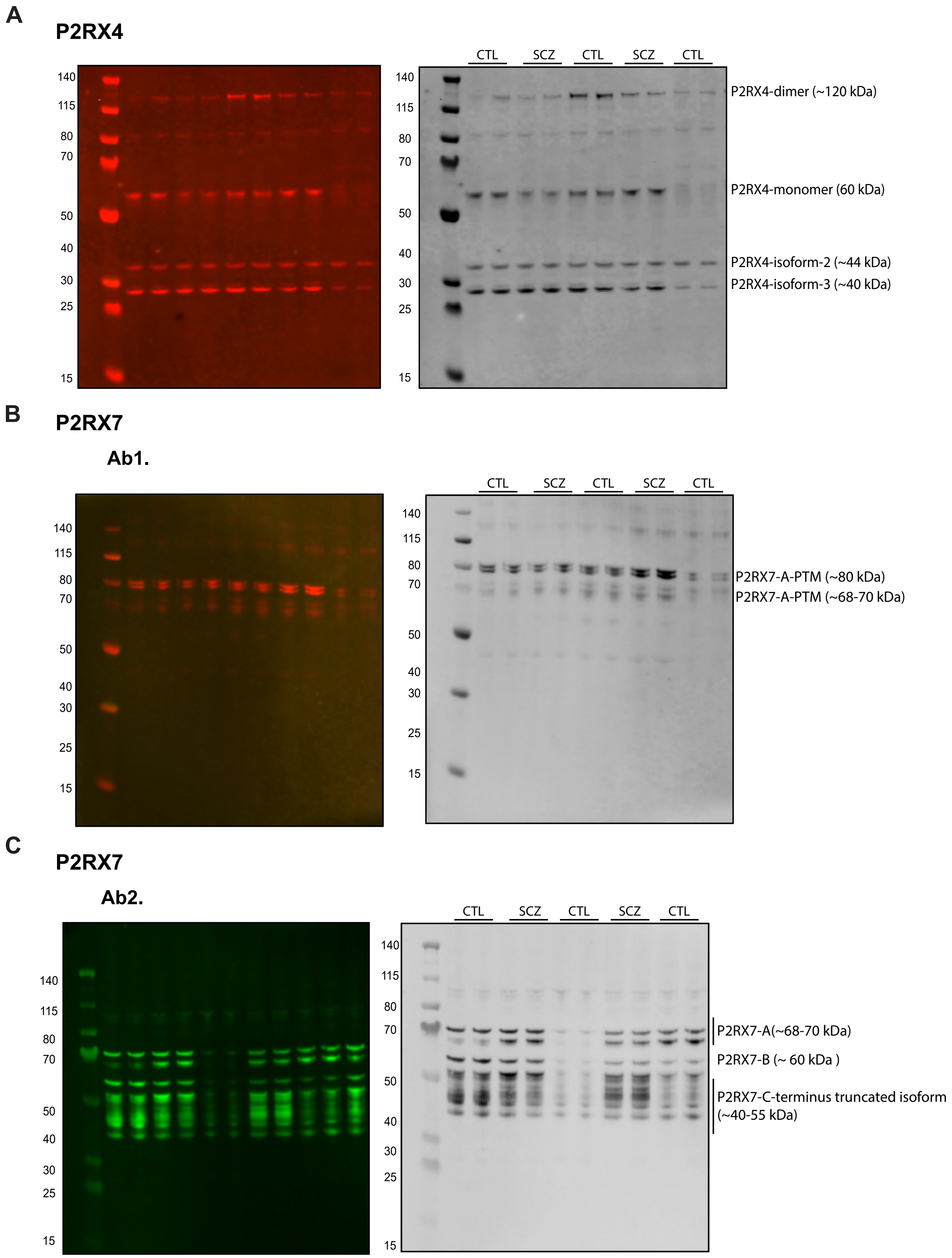

Figure S4. Representative Western immunoblot analysis of P2RX protein expression in postmortem human brain tissue. CTL and SCZ samples were run in duplicate for each subject. Proteins were quantified by infrared imaging with fluorescent secondary antibodies. (A) Immunoblot for P2RX4 using knockout validated antibody. (B) Immunoblot for P2RX7 using knockout validated antibody (Ab1). (C) Immunoblot for N-terminus directed antibody (Ab2).

DLPFC cohort								
DX	AGE	RACE	SEX	pH	PMI	ON/OFF antipsychotic medication	Antipsychotic drug class	Tissue used in experiments
CTL	32	W	M	N/A	10			
CTL	23	W	F	6.54	12			
CTL	49	W	M	N/A	8			
CTL	41	W	M	N/A	10			
CTL	44	B	M	N/A	9			
CTL	57	W	F	6.6	22			
CTL	47	W	M	6.6	6			
CTL	32	W	F	7.37	7			
CTL	48	B	F	6.23	12			
CTL	42	W	F	6.21	12			
CTL	45	W	F	5.74	12			qPCR
CTL	55	W	M	6.87	10			
CTL	31	W	F	6.66	21			
CTL	45	W	F	6.5	16			
CTL	41	B	M	6.68	14			
CTL	47	B	M	6.39	14			qPCR
CTL	28	W	M	6.30	13			
CTL	40	B	M	6.68	7			
CTL	37	W	F	6.49	10			
CTL	55	B	F	6.8	24			
CTL	45	B	M	N/A	16			WB
CTL	54	B	M	6.88	20			WB
CTL	45	W	M	6.97	18			WB
CTL	49	B	M	6.76	19			WB
SCZ	35	W	M	6.07	7	OFF		
SCZ	45	B	M	7.1	10	Unknown	Unknown	qPCR
SCZ	54	W	M	6.61	12	OFF		
SCZ	39	W	F	N/A	24	Unknown	Unknown	
SCZ	44	B	M	6.35	6	ON	Typical	
SCZ	57	B	M	6.43	9	ON	Typical	
SCZ	67	B	M	6.36	8	ON	Typical	
SCZ	32	B	F	6.31	12	OFF		
SCZ	45	B	F	6.2	10	Unknown	Unknown	
SCZ	34	W	M	6.75	16	Unknown	Unknown	
SCZ	48	W	F	7.8	21	Unknown	Unknown	
SCZ	43	W	F	6.91	8	ON	Atypical	qPCR
SCZ	34	W	F	6.56	27	Unknown	Unknown	
SCZ	47	W	M	6.47	16	ON	Atypical	qPCR
SCZ	41	B	F	N/A	19	ON	Atypical	
SCZ	55	W	M	6.35	12	Unknown	Unknown	

SCZ	42	W	F	6.8	14	Unknown	Unknown	
SCZ	56	W	F	N/A	20	Unknown	Unknown	
SCZ	33	W	F	N/A	16	Unknown	Unknown	
SCZ	42	W	M	N/A	6	Unknown	Unknown	
SCZ	45	B	M	6.66	16	ON	Typical	WB
SCZ	42	B	M	7.1	16	Unknown	Unknown	WB
SCZ	42	B	M	6.8	20	ON	Typical	WB
SCZ	20	W	M	6.66	23	OFF		WB
SCZ	56	B	M	7.12	25	Unknown	Unknown	WB

ACC cohort					
**DX**	**AGE**	**SEX**	**pH**	**PMI**	**ON/OFF antipsychotic medication**
CTL	78	M	N/A	8.1	N/A
CTL	78	F	6.48	4.3	N/A
CTL	79	F	5.72	7.2	N/A
CTL	86	F	N/A	11.5	N/A
CTL	78	M	N/A	5.9	N/A
CTL	85	M	N/A	16.2	N/A
CTL	64	M	6.12	10.4	N/A
CTL	85	F	7.27	8.0	N/A
CTL	68	F	6.30	24.0	N/A
CTL	71	M	N/A	5.6	N/A
CTL	75	F	N/A	3.3	N/A
CTL	84	M	6.62	20.9	N/A
CTL	71	M	7.09	21.7	N/A
CTL	73	M	6.94	21.1	N/A
CTL	86	F	N/A	10.2	N/A
CTL	71	M	N/A	21.4	N/A
CTL	79	M	N/A	16.1	N/A
CTL	85	M	N/A	5.3	N/A
CTL	84	M	N/A	16.8	N/A
CTL	85	F	N/A	5.3	N/A
SCZ	70	F	6.21	13.9	Unknown
SCZ	74	F	6.3	7.0	ON
SCZ	81	F	5.93	12.5	OFF
SCZ	82	F	5.89	8.8	OFF
SCZ	86	F	5.8	18.2	ON
SCZ	73	M	6.15	8.8	ON
SCZ	90	F	5.97	7.8	ON
SCZ	77	M	6.4	24.0	OFF
SCZ	73	M	6.35	7.2	ON
SCZ	70	M	6.49	14.3	ON

SCZ	68	M	6.27	8.9	ON
SCZ	86	M	6.48	15.4	ON
SCZ	75	M	5.85	5.8	ON
SCZ	81	F	6.47	15.1	OFF
SCZ	70	M	6.36	17.3	OFF
SCZ	75	F	6.49	21.5	Unknown
SCZ	62	F	6.74	23.7	ON
SCZ	71	M	N/A	9.5	ON
SCZ	83	M	N/A	16.3	OFF
SCZ	61	M	N/A	6.2	ON

Table S1: Individual subject demographics for dorsolateral prefrontal cortex (DLPFC) obtained from the Maryland Brain Collection (MBC) and anterior cingulate cortex (ACC) obtained from the NIH Brain and Tissue Repository (NBTR) brain regions. Tissue from the subjects was used in all experiments unless otherwise indicated. qPCR quantitative polymerase chain reaction; WB western blot.

B black; CTL control; DX diagnosis; F female; M male; N/A not applicable; PMI (hrs) postmortem interval in hours; qPCR quantitative polymerase chain reaction; SCZ schizophrenia; W white; WB western blot.

	ACC			
	Female CTL	Female SCZ	Male CTL	Male SCZ
**N**	8	9	12	11
**Age (mean±SD)**	80.25 ± 6.50	77.89 ± 8.56	76.92 ± 6.90	73.36 ± 6.91
**pH**	6.44 ± 0.64	6.13 ± 0.320	6.69 ± 0.429	6.35 ±0.11
**PMI (hrs)**	9.2 ± 6.60	14.3 ± 5.98	14.12 ± 6.66	12.1 ± 5.74
	DLPFC qPCR cohort			
	Female CTL	Female SCZ	Male CTL	Male SCZ
**N**	10	10	10	10
**Age (mean±SD)**	41.5 ± 10.81	41.3 ± 7.39	42.40 ± 7.97	48 ± 10.29
**pH**	6.51 ± 0.42	6.76 ± 0.57	6.59 ± 0.20	6.50 ± 0.29
**PMI (hrs)**	14.8 ± 5.69	17.1 ± 6.17	10.10 ± 2.80	10.2 ± 3.73
	DLPFC Western blot cohort			
	Female CTL	Female SCZ	Male CTL	Male SCZ
**N**	9	9	13	13
**Age (mean±SD)**	41.11 ± 11.39	41.22 ± 7.71	43.84 ± 7.72	45.61 ± 12.24
**pH**	6.60 ± 0.34	6.73 ± 0.63	6.72 ± 0.20	6.61 ± 0.31
**PMI (hrs)**	15.11 ± 5.94	18.11 ± 5.60	12.31 ± 4.71	13.54 ± 6.41

Table S2. Summary subject demographics by sex and diagnosis. DLPFC dorsolateral prefrontal cortex; ACC anterior cingulate cortex; CTL control; SCZ schizophrenia; PMI (hrs) postmortem interval in hours; N subject number. Data presented as mean +/- standard deviation.

**Human qPCR primers**			
Gene	**Forward Sequence**	**Reverse Sequence**	**Citation**
		**SYBR-Green primers**	
P2RX4	AGATGCGACCACTGTGTGTA	GTTGAGACTCCGTTGCTGTG	[1]
P2RX5	TTCACCAACACCTCGGATCT	CAGGTTGGTGACCACAAAAA	[2]
P2RX7	TGTCCCATTTTCCGACTAGG	CCAACGGTCTAGGTTGCAGT	[1]
P2RY12	GGTAACCAACAAGAAATGCAAGC	GGACAGTGTAGAGCAGTGGGAAG	[3]
P2RY13	ATCGTGCTGTTAGGGCTCATA	CAAGATCGTATTTGGCAGGGAG	[4]
B2M	GTGGGATCGAGACATGTAAGC	AGCAAGCAAGCAGAATTTGGAAT	[5]
GAPDH	TCGACAGTCAGCCGCATCT	AGTTAAAAGCAGCCCTGGTGA	[6]
ACTB	GTCATTCCAAATATGAGATGCGT	GCTATCACCTCCCCTGTGTG	[7]
PPIA	ATGGTCAACCCCACCGTGTTCTTCG	CGTGTGAAGTCACCACCCTGACACA	[8]
		**Taqman primers**	
SERPINA3		Hs00153674_m1	n/a
GAPDH		Hs99999905_m1	n/a
B2M		Hs99999907_m1	n/a
PPIA		Hs99999904_m1	n/a

**Rat qPCR primers**			
Gene	**Forward Sequence**	**Reverse Sequence**	**Citation**
P2rx4	AGATGCGACCACTGTGTGTA	GTTGAGACTCCGTTGCTGTG	[1]
P2rx7	TGTCCCATTTTCCGACTAGG	CCAACGGTCTAGGTTGCAGT	[1]
P2rx5	CATCATCCCCACAGTCATCA	AGTACCAGGTCGCAGAAGAAAG	[9]
18s	CGCCGCTAGAGGTGAAATTC	TTGGCAAATGCTTTCGCTC	[5]
Gapdh	AGCCCAGAACATCATCCCTG	CACCACCTTCTTGATGTCATC	[10]
Actb	AGTACTCTGTGTGGATTGGT	GCTGATCCACATCTGCTGGA	[5]
Ppia	CTCCTTTGAGCTGTTTGCAG	CACCACATGCTTGCCATCC	[5]

Table S3. Primer sequences for human and rat P2 receptor and reference gene targets. Primer sequences as previously published. Taqman primers selected from manufacturer.

References

1 Walenta, L. *et al.* ATP-mediated Events in Peritubular Cells Contribute to Sterile Testicular Inflammation. *Sci Rep* **8**, 1431, doi:10.1038/s41598-018-19624-3 (2018).

2 Rockstroh, D. *et al.* Direct evidence of brown adipocytes in different fat depots in children. *PloS one* **10**, e0117841, doi:10.1371/journal.pone.0117841 (2015).

3 Li, M. *et al.* Associations between P2RY12 gene polymorphisms and risks of clopidogrel resistance and adverse cardiovascular events after PCI in patients with acute coronary syndrome. *Medicine (Baltimore)* **96**, e6553, doi:10.1097/MD.0000000000006553 (2017).

4 Wang, W., Yi, X., Ren, Y. & Xie, Q. Effects of Adenosine Triphosphate on Proliferation and Odontoblastic Differentiation of Human Dental Pulp Cells. *J Endod* **42**, 1483-1489, doi:10.1016/j.joen.2016.07.013 (2016).

5 O'Donovan, S. M. *et al.* Glutamate transporter splice variant expression in an enriched pyramidal cell population in schizophrenia. *Translational psychiatry* **5**, e579, doi:10.1038/tp.2015.74 (2015).

6 Cai, P. *et al.* A genome-wide long noncoding RNA CRISPRi screen identifies PRANCR as a novel regulator of epidermal homeostasis. *Genome research* **30**, 22-34, doi:10.1101/gr.251561.119
gr.251561.119 [pii] (2020).

7 Zhou, Z. *et al.* MicroRNA-27a promotes podocyte injury via PPARgamma-mediated beta-catenin activation in diabetic nephropathy. *Cell Death Dis* **8**, e2658, doi:10.1038/cddis.2017.74
cddis201774 [pii] (2017).

8 Dean, B., Udawela, M. & Scarr, E. Validating reference genes using minimally transformed qpcr data: findings in human cortex and outcomes in schizophrenia. *BMC psychiatry* **16**, 154, doi:10.1186/s12888-016-0855-0
10.1186/s12888-016-0855-0 [pii] (2016).

9 McClelland, S. *et al.* The transcription factor NRSF contributes to epileptogenesis by selective repression of a subset of target genes. *Elife* **3**, e01267, doi:10.7554/eLife.01267 (2014).

10 Jang, Y. O. *et al.* Effect of bone marrow-derived mesenchymal stem cells on hepatic fibrosis in a thioacetamide-induced cirrhotic rat model. *BMC Gastroenterol* **14**, 198, doi:10.1186/s12876-014-0198-6
s12876-014-0198-6 [pii] (2014).
